# The Calcitonin and Glucocorticoids Combination: Mechanistic Insights into Their Class–Effect Synergy in Experimental Arthritis

**DOI:** 10.1371/journal.pone.0054299

**Published:** 2013-02-05

**Authors:** Adam Al-Kashi, Trinidad Montero-Melendez, Niloufar Moradi-Bidhendi, James P. Gilligan, Nozer Mehta, Mauro Perretti

**Affiliations:** 1 The William Harvey Research Institute, Barts and The London School of Medicine, London, United Kingdom; 2 Tarsa Therapeutics, Philadelphia, Pennsylvania, United States of America; 3 Unigene Corporation, Fairfield, New Jersey, United States of America; McMaster University, Canada

## Abstract

**Introduction:**

Previous work reported the anti-arthritic synergy afforded by combining calcitonin (CT) and glucocorticoids (GC). Here we focus on the pairing of elcatonin (eCT) and dexamethasone (Dex), querying whether: i) this was a class-effect action; ii) mechanistic insights could be unveiled; iii) the synergy affected canonical GC adverse effects.

**Methods:**

Using the rat collagen-induced arthritis model, different combinations of eCT and Dex, were administered from disease onset to peak (day 11 to 18). Macroscopic disease score was monitored throughout, with biochemical and histological analyses conducted on plasma and tissues at day 18. The effect on acute hyperglycaemia and liver enzyme message were also assessed.

**Results:**

Whilst eCT alone was inactive, it synergised at 1 µg/kg with low doses of Dex (7.5 or 15 µg/kg) to yield an anti-arthritic efficacy equivalent to a 4- to 7-fold higher Dex dose. Mechanistically, the anti-arthritic synergy corresponded to a marked attenuation in RA-relevant analytes. CXCL5 expression, in both plasma and joint, was markedly inhibited by the co-therapy. Finally, co-administration of eCT did not exacerbate metrics of GC adverse effects, and rescued some of them.

**Conclusions:**

We present evidence of a class-effect action for the anti-arthritic synergy of CT/GC combination, underpinned by the powerful inhibition of joint destruction markers. Furthermore, we identify CXCL5 as a marker for the combination therapy with potential diagnostic and prognostic utility. Substantial GC dose reduction, together with the absence of exacerbated adverse effects, indicated a significant clinical potential for this co-therapy in RA and beyond.

## Introduction

Glucocorticoids (GCs) are fundamental therapeutics in the treatment of inflammatory diseases. Their clinical benefits derive from a complex spectrum of effects downstream of GC receptor (GR) activation, which serves to modulate several thousand genes amounting to ∼1% of the genome [1]. This results in the down-regulation of many pro-inflammatory mediators [2], [3] and up-regulation of anti-inflammatory and pro-resolving factors [2]. However, these benefits come at a price: a veritable catalogue of adverse effects, particularly evident upon mid- to long-term administration. Amongst the more severe of these are hyperglycaemia, osteoporosis and hypertension [4]–[6]. Despite these adverse effects, the use of GCs in the treatment of inflammatory diseases has remained widespread. The juxtaposition between benefit and detriment justifies pre-clinical efforts to identify better treatment regimens.

There has been a significant research effort focused on the development of GCs exhibiting an improved pharmacological profile, retaining anti-inflammatory efficacy with reduced adverse effects [7]. Selective GR agonists or ‘dissociated’ steroids may offer a more pronounced transrepression over transactivation, resulting in fewer adverse effects [8]. Unfortunately, despite early optimism [9], dissociated steroids have thus far failed to translate smoothly from preclinical to clinical investigations [10], and, since transactivation of anti-inflammatory and pro-resolving factors represents a relevant part of GC efficacy [11], it is perhaps of little wonder. In recent years, a modified-release prednisone formulation has demonstrated significantly increased efficacy in the attenuation of RA morning stiffness, without changing the safety profile [12]. Previous pharmacokinetic strategies have included the use of alternate routes of administration to avoid systemic exposure [13] and the development of shorter half-life GCs with a similar aim [14]. Low-dose GC therapy appears to provide a degree of benefit with only modest adverse effects [15], and the recent EULAR recommendations for cardiovascular risk management in RA patients support the approach of minimal dose for minimal duration [16]. However, reducing exposure, and consequent efficacy, in a bid to avoid adverse effects serves as an apt reminder of our failure to adequately separate GC-induced benefit from GC-induced harm.

Calcitonin (CT) is a 32-amino acid peptide secreted by the parafollicular cells of the mammalian thyroid, and by the ultimobrachial body of many lower vertebrates. It was discovered in 1961 as a hypocalcaemic hormone [17], and mechanistic insights soon followed, with observations of increased urinary calcium and reduced urinary hydroxyproline (a bone resorption marker) [18]. The anti-resorptive effect was soon exploited therapeutically in the treatment of bone metabolic disorders [19], and is now understood to involve a direct effect upon the osteoclast, blocking reactivity to several activators, including RANKL and GC [20], [21]. CT can exert a direct protective effect on chondrocytes, enhancing collagen and proteoglycan synthesis with beneficial implications for diseases involving cartilage degradation [22], [23]. It also displays modulatory functions on other RA-relevant cells, including osteoblasts [24] and perhaps synoviocytes [25]. Decades on from its discovery, CT is a standard therapeutic option for Paget’s disease of the bone, and also effective against post-menopausal- and GC-induced osteoporosis [26], but its near exclusive identification with bone protection belies under-exploited potentials, particularly in anti-inflammation and analgesia.

Anti-inflammatory effects for CT have been demonstrated in a number of animal models, including adjuvant-induced arthritis [27], though not in collagen-induced arthritis [28]. Human lymphocytes express CT receptors which are regulated by IL-1 and IL-6 [29], suggesting an immunomodulatory role, and CT treatment *in vitro* has been found to reduce intracellular and secreted IL-1α/β in leukocytes from RA patients [30].

Collagen-induced arthritis (CIA) in the rat shares many similarities with human RA, including synovial hyperplasia, immune cell infiltration and marginal bone erosions [31]. Unlike the adjuvant-induced model, which is associated with non-relevant extra-articular manifestations, CIA also results in the generation of rheumatoid factor [31]–[32]. Using the rat CIA model, we previously reported that the co-administration of salmon CT and prednisolone produced an unexpected anti-arthritic synergism, affording GC dose reduction [20]. The applicability of this co-treatment to other GCs, as well as the molecular mechanisms that underlie these synergies, were not clarified. The present study was undertaken in order to broaden our knowledge of the CT/GC combination. Particular emphases were placed on further characterisation, mechanistic inquiry and a survey of classical GC adverse effects. We have expanded the previous molecule-specific observations to a class effect action through the use of an alternate CT/GC pairing, namely elcatonin (eCT) and dexamethasone (Dex), and propose the clinical testing of this synergy.

## Materials and Methods

Materials were purchased from Sigma-Aldrich Co. (Poole, UK) unless otherwise specified. Elcatonin (eCT) was purchased from Bachem UK Ltd. (Merseyside, UK). Bovine nasal collagen-II (CII) was purified in-house from source material.

### Collagen-induced Arthritis

Female Lewis rats (150±20 g body weight; Harlan UK Ltd., Bicester, UK) were fed a standard chow pellet and water diet *ad libitum*, and maintained on a 12-hour light/dark cycle. Animal work was conducted under license from the Home Office and in accordance with the Animals (Scientific Procedures) Act, 1986.

Bovine CII was dissolved in acetic acid (0.01 M) at 4 mg/ml and emulsified in an equal volume of complete Freund’s adjuvant (CFA). On day 0, rats were anaesthetized with isoflurane and then injected intradermally with 200 µl of the CII/CFA emulsion (400 µg of collagen-II per rat) at the base of the tail. Elcatonin was dissolved in PBS with 0.1% bovine serum albumin (BSA). Dex was pre-solubilised in dimethyl sulphoxide (DMSO) prior to diluting in PBS with 0.1% BSA (final DMSO concentration was less than 0.05% and considered negligible). Elcatonin, Dex and combinations thereof were given daily by intraperitoneal injection from day 11 (typical arthritis onset) onwards. Routinely, groups of 8 rats for each treatment were used (range from 7 to 10). Hind paw volume measurements were quantified by water displacement (Ugo Basile, Milan, Italy) whilst clinical scores were recorded using a three-point scale of anatomical region involvement, with ankle, pad and digits each contributing one point (giving a maximum score of 6). On day 18 (or day 21 in preliminary experiments), animals were killed by cervical dislocation and blood was collected by cardiac puncture on to heparin (50 U/ml of blood; Leo Pharma, Buckinghamshire, UK) and plasma prepared by centrifugation. Hind paws, liver tissue and plasma aliquots were stored at −80°C unless being processed for immediate analyses.

### Histological Processing and Assessment

Hind paws were fixed overnight in 4% neutral buffered paraformaldehyde, decalcified for 7–10 days in 30% formic acid with 0.5M trisodium citrate, and embedded in paraffin. Longitudinal sections (5 µm) were cut from the centre of the ankle joint in the sagittal plane and stained with haematoxylin-eosin. Sections were examined by light microscopy for cellular infiltration, synovitis, bone erosion and structural integrity.

### Preparation of Paw Tissue Extracts

Hind paws were homogenized whole (Wet n’ Dry Grinder, Revel, Houston, Texas) in 5 ml PBS with 1% Triton X100 and protease inhibitors (complete EDTA-free protease inhibitor tablets; Roche Diagnostics, West Sussex, UK). After 1-hour incubation at room temperature, supernatants were collected by centrifugation. Samples were normalised by total protein concentration (BCA protein assay; Thermo Scientific Pierce, Loughborough, UK).

### Immunochemical Assays

The following markers were assayed using commercially available kits: MMP-2, CXCL5, CCL5, CCL20, CXCL7 ELISA from R&D Systems (Abingdon, UK); telopeptide of type-I collagen (CTX-I) EIA from Immunodiagnostic Systems (Tyne & Wear, UK); TRAP-5b and ACTH EIA from Biosupply UK Ltd. (Bradford, UK). Circulating levels of serum amyloid protein A (SAA) were quantified by ELISA (TSZ ELISA, Framingham, Maryland, USA). Proteome profiling was performed by rat cytokine array kit from R&D Systems.

### Assessment of GC Induced Off-target Side Effects

Rats (routinely 8 animals per treatment) were fasted for 14 h and then given a single dose of Dex (15 or 100 µg/kg i.p.) with or without 1.0 µg/kg eCT. Blood was collected by venepuncture and glucose was quantified immediately prior to fasting, prior to drug treatment, and 5 h after drug treatment by Accu-Chek meter (Roche Diagnostics, West Sussex, UK). Blood was then collected by cardiac puncture on to EDTA (2 mM) for adrenocorticotropin (ACTH) assay, and animals were killed by cervical dislocation. Liver tissue was collected and stored at −80°C.

Quantitative real-time PCR was performed in liver tissue from the CIA and hyperglycaemia experiments (routinely 4 samples per treatment were analysed). RNA was extracted using the RNeasy Mini Plus Kit from Qiagen UK Ltd. (Crawley, UK). cDNA was synthesized using 1-µg of total RNA with the SuperScript III Reverse Transcriptase (Invitrogen, Paisley UK). Real time-PCR was performed with 200-ng of cDNA per well and Power SYBR Green PCR Master Mix (Applied Biosystems, Warrington, UK), using the ABI Prism 7900HT Sequence Detection System, and commercially available primers for tyrosine aminotransferase (Tat; QT00182308), phosphoenolpyruvate carboxykinase (Pck2; QT01825327), glucose-6-phosphatase (G6pc3; QT00190610) and fructose-1,6-bisphosphase (Fbp2, QT01791076); all from Qiagen UK Ltd. Glyceraldehyde 3-phosphate dehydrogenase mRNA (Gapdh, QT00199633) was used as internal control. Data was expressed as 2^−ΔΔCt^, where ΔCt = Ct of the target gene (e.g.Tat) – Ct of the internal control gene (Gapdh), and ΔΔCt = ΔCt of the samples for target gene – ΔCt of the calibrator (control group) for the target gene.

### Statistics

Data are presented as mean ± SEM of *n* number of rats. Data analyses were conducted by one-way ANOVA comprising Kruskal-Wallis test and Dunn’s post-test for multiple comparisons, or by Mann-Whitney U test for single comparisons; both with an alpha value of p>0.05.

## Results

### Rat CIA is Highly Sensitive to Dex

**Figure 1:**
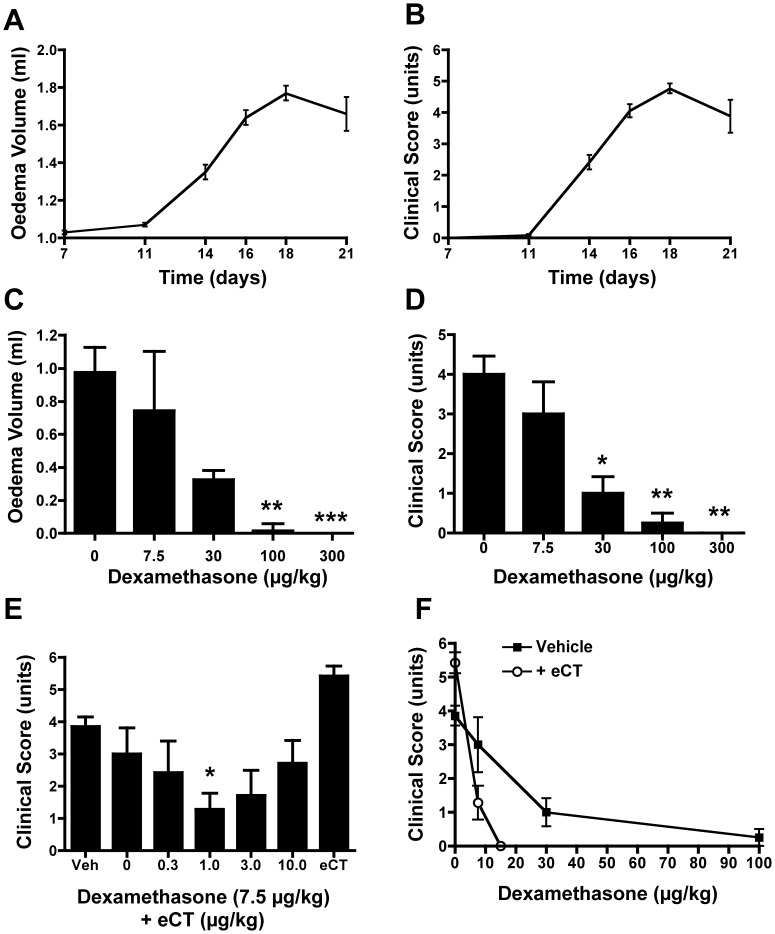
Elcatonin synergises with Dexamethasone in the rat CIA model. (A, B) Time course of CIA in rats. Collagen was given at Day 0 and arthritis developed from Day 11 and peaked at Day 18, as measured by paw oedema volume (A) and clinical score (B). Data, mean ± SEM, are cumulative of twelve CIA experiments (n = 76 to 82 rats, except for day 21 where n = 16). (C, D) In separate experiments, at first signs of disease (Day 11), vehicle or Dexamethasone (Dex) were given daily i.p. with powerful inhibition of hind paw oedema (C) and clinical score (D), as shown for peak response at Day 18. Data are mean ± SEM (n = 4 to 8 rats). (E) Having established the glucocorticoid dose response, a sub-therapeutic dose of Dexamethasone (7.5 µg/kg) was combined with elcatonin (eCT; 0–10 µg/kg) – given i.p. from Day 11 - revealing an anti-arthritic synergy, shown here at peak with Day 18 values. Treatment with elcatonin (1 µg/kg) alone is alone shown (eCT). Data are mean ± SEM of 10 rats. (F) Co-administration of 1.0 µg/kg eCT shifts Dexamethasone dose-response curve to the left (both compounds given from i.p. from Day 11), enabling abolition of clinical score presentation with an 85% lower dose, see Dex 15 µg/kg versus 100 µg/kg (all values are from peak, Day 18). Data are mean ± SEM of 10 animals. In all cases, statistical analyses by one-way ANOVA (Kruskal-Wallis test with Dunn’s post-test); *p<0.05, **p<0.01, and ***p<0.001 as compared to vehicle-treated group (dose 0).

Paw volume data, pooled from twelve separate experiments, clearly illustrates the time course of the CIA reaction (Figure 1A), with mean onset at day 11 and mean peak at day 18. Clinical scores displayed an identical time course when viewed across the entire study (Figure 1B). Disease incidence for this protocol was ∼100% at the reaction peak (Figure S1). Dex is potently anti-arthritic in the rat CIA model, with abrogation of paw swelling and clinical score at the 100 µg/kg dose given from day 11 onwards (Figure 1C/D). Sub-therapeutic and moderately therapeutic doses of 7.5 and 30 µg/kg, respectively, were used in subsequent experiments.

### Elcatonin Affords GC Dose Reduction Through Anti-arthritic Synergy

The synergistic CT-GC interaction has been evidenced previously in this lab, using salmon calcitonin and prednisolone in the rat CIA model [20]. One of the aims of this present study was to expand this agent-specific observation to a class-effect one. Thus, having characterised the efficacy of Dex, we next endeavoured to find the optimal eCT dose for co-administration, testing the 0.3–10 µg/kg range, given daily from day 11. Figure S2 depicts a representative time-course experiment. The result was an inverse bell-shaped relationship that centred on the optimal dose of 1.0 µg/kg eCT (Figure 1E). The combination of sub-therapeutic Dex (7.5 µg/kg) with eCT resulted in approximate 68% attenuation of clinical score, which was not significantly different from the 75% attenuation achieved by 30 µg/kg Dex (Figure 1D). Thus, this synergy affords a 4-fold Dex dose reduction.

We then undertook one further step of co-treatment optimisation by testing a ‘borderline’ therapeutic dose of Dex (15 µg/kg). Co-therapy with eCT (1.0 µg/kg) from day 11 resulted in abolition of clinical score presentation, akin to a 7-fold higher dose of Dex alone (Figure 1F).

### Histological Analysis Correlates to Macroscopic Efficacy

**Figure 2:**
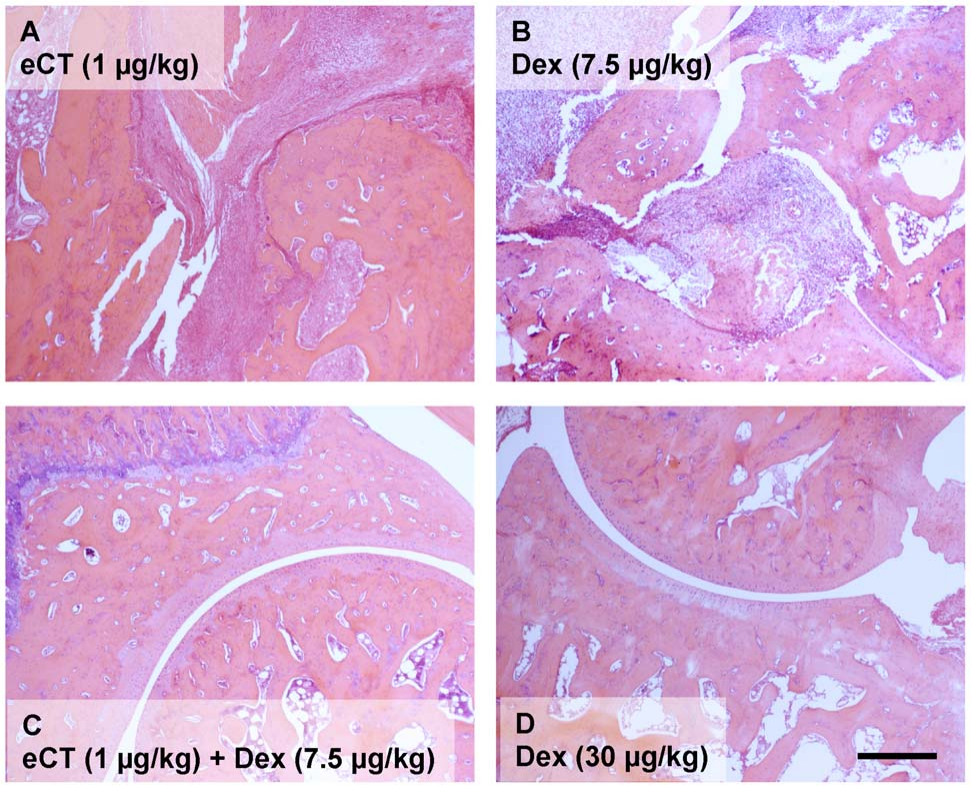
Combination of elcatonin and sub-therapeutic Dexamethasone preserves articular integrity. Day 18 CIA rat hind paws from rats treated with collagen at Day 0. Elcatonin (eCT) and Dexamethasone (Dex) were given i.p. daily from Day 11, as detailed in Legend to Figure 1. Paws were fixed, de-calcified, paraffin-embedded, sectioned and stained with haematoxylin-eosin. Light micrographs (representative of 4 animals), showing synovitis, cellular infiltrate, bone erosion and articular integrity. (A) eCT 1.0 µg/kg alone, (B) Dex 7.5 µg/kg alone, (C) co-treatment: eCT 1.0 µg/kg+Dex 7.5 µg/kg, (D) Dex 30 µg/kg. Scale bar, 300 µm.

Microscopic examination of the tarsal region revealed a clear distinction between therapeutic and non-therapeutic regimens. Joints from vehicle-treated animals showed intense cellular infiltration and synovitis with focal bone erosions and distorted joint architecture (not shown), which was left unchanged by either eCT or sub-therapeutic Dex alone (Figure 2A, B). In contrast, the co-therapy regimen left structurally joints intact with minimal or no infiltrate (Figure 2C), in line with what observed with full dose Dex monotherapy (Figure 2D), which yield a histological image not distinguishable from that of naïve joints.

### Biochemical Markers in Tissue and Plasma

**Figure 3:**
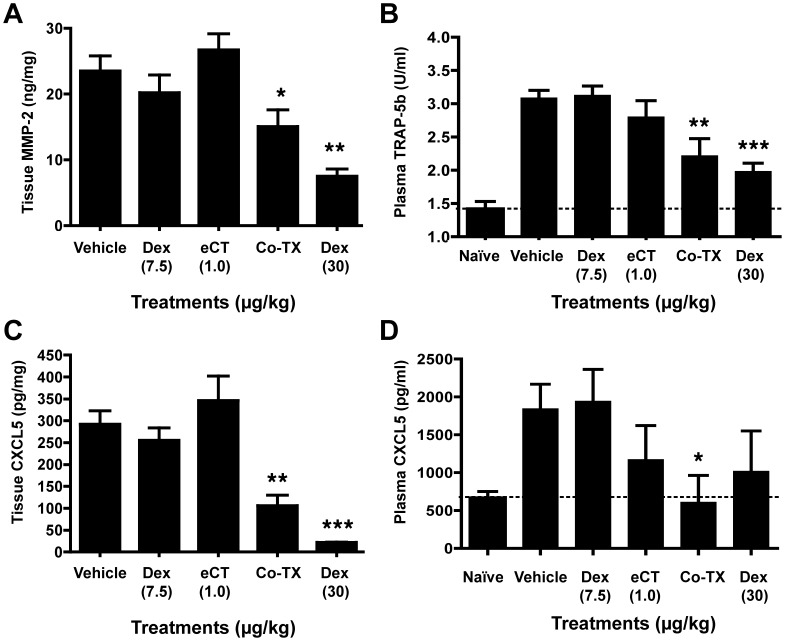
The combination elcatonin/Dexamethasone elicits a synergistic attenuation of MMP-2, TRAP-5b and CXCL5 expression. Rats were treated with collagen on Day 0 and then, from Day 11, received daily i.p. injections of elcatonin (eCT; 1.0 µg/kg) alone or together with a sub-therapeutic dose of Dexamethasone (D; 7.5 µg/kg) (Co-Tx, combination therapy). A positive control group of rats was treated with Dexamethasone (30 µg/kg). In all cases hind paw tissue extracts and plasma samples were taken at day 18. Protein levels of (A) metalloproteinase II (MMP-2) in tissue extracts, (B) plasma tartrate-resistant acid phosphatase (TRAP-5b), (C) tissue extract and (D) plasma CXCL5 were determined as described in Methods. Data are mean ± SEM of 10 rats per group. Statistical analyses by one-way ANOVA (Kruskal-Wallis test with Dunn’s post-test); *p<0.05, **p<0.01, and ***p<0.001 as compared to vehicle-treated group.

Having established and optimised drug doses for the co-treatment, we next investigated the biochemical correlates of the anti-arthritic efficacy, starting with classical markers. Analysis of total matrix metalloproteinase (MMP)-2 levels in hind paw tissue extracts revealed a significant attenuation by the co-administration of eCT with sub-therapeutic Dex, but not by the individual therapies alone (Figure 3A). Meanwhile, serum C-terminal cross-linking telopeptide of type-I collagen (CTX-I) – a classical circulating and urinary marker of bone resorption [33] – was significantly reduced from 24.2±2.0 ng/ml in vehicle-treated animals to 12.4±3.7 and 11.1±2.3 ng/ml in animals given eCT alone or with sub-therapeutic Dex, respectively (n = 7 to 14; P<0.01 in either case), i.e. displaying no synergism. Plasma TRAP-5b more than doubled in rat CIA compared to the plasma of naïve counterparts (Figure 3B). This pathology-associated elevation in TRAP-5b was significantly reduced in the co-therapy treated animals, but not in those receiving either eCT or sub-therapeutic Dex alone (Figure 3B).

The above markers are known to be associated with joint disease/cells and clearly their modulation by eCT ± Dex is of importance. In order to expand the field of knowledge, we employed a proteome-profiling assay to screen for unpredicted markers for the co-therapy. The dot blot-like cytokine assay was run on joints extracts yielding several semi-quantitative hits (Figure S3). Four chemokines were taken forward for precise quantification, however for three of them we did not observe significant modulation, namely CCL5, CCL20 or CXCL7 (Figure S4). In contrast, tissue CXCL5 was reduced by 64% in response to the CT/GC co-therapy (Figure 3C). Analysis of plasma CXCL5 revealed that circulating levels of this chemokine are increased in arthritic rats, compared to naïve animals, and that this increase is abolished by the co-therapy (Figure 3D). Both for tissue and plasma CXCL5 values, low dose Dex or eCT alone were inactive, whilst significant reduction were attained by the co-therapy.

Circulating levels of SAA were also measured, observing a ∼25% reduction in the Dex (7.5 µg/kg group) compared to vehicle-treated arthritic rats (3804±266 *vs.* 4916±195 U/ml; n = 8; P<0.05). This effect was not altered by the co-treatment with eCT (1 µg/kg; SAA values of 3766±246; not significant from Dex alone).

### Assessing the Impact of eCT Co-administration on Classical GC Adverse Effects

In light of the profound therapeutic enhancement afforded by eCT co-administration, it became relevant to assess if the co-therapy also augmented classical GC adverse effects.

**Figure 4:**
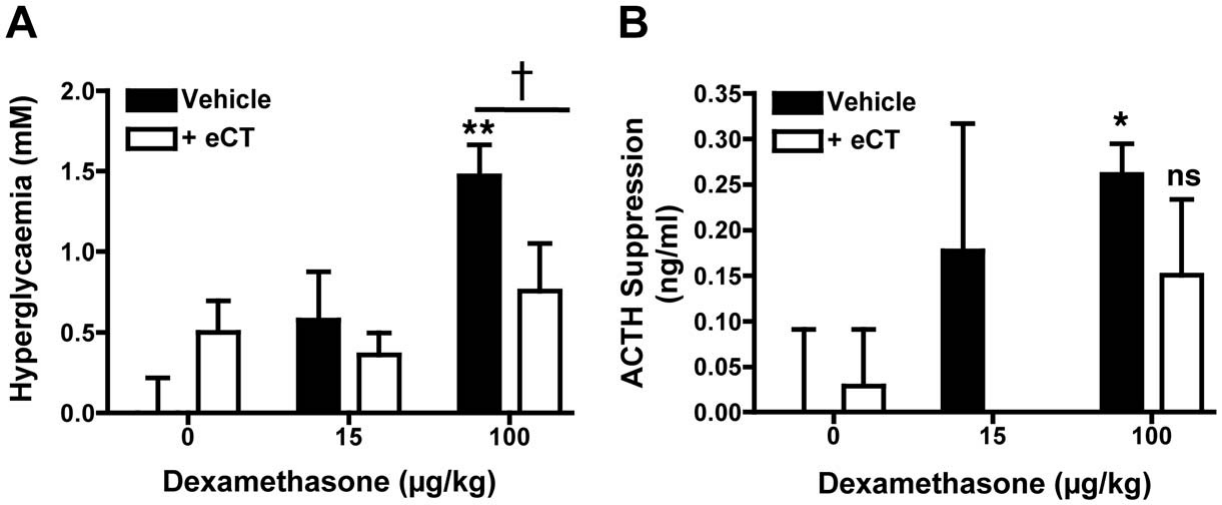
Elcatonin does not augment Dexamethasone-induced hyperglycaemia and ACTH suppression. Fasted rats were given a single dose of Dexamethasone (15 or 100 µg/kg i.p.) with or without 1.0 µg/kg elcatonin (eCT). Blood was collected by venepuncture and glucose was quantified: i) immediately prior to fasting, ii) prior to drug treatment, and iii) 5 h after drug treatment. Overnight fasting caused a fall in mean blood glucose from 6.19±0.10 to 4.80±0.13 mM. Blood glucose in the vehicle-treated group continued to drop, reaching 3.86±0.22 mM at the 5-hour time point. (A) Blood glucose data as measured 5 h post-treatment, and normalised for differing pre-fasting levels. (B) ACTH was assayed by EIA in blood collected by terminal cardiac puncture at 5 h post-treatment. ACTH suppression is expressed in relation to the levels quantified by ELISA in vehicle-treated animals (1.57±0.09 ng/ml). In both panels, data are mean ± SEM of 8 rats per group. Statistical analyses by one-way ANOVA (Kruskal-Wallis test with Dunn’s post-test); *p<0.05, **p<0.01, and ***p<0.001 as compared to vehicle-treated group, or by Mann-Whitney test; ^†^p>0.05 in the indicated comparison.

Elcatonin co-administration was assessed using a protocol of acute GC-induced hyperglycaemia. Rats were fasted overnight prior to receiving a single dose of Dex with or without eCT. Blood glucose was assessed for hyperglycaemia after five hours. As reported above (Figure 1F), the optimal co-therapy was as efficacious as high-dose Dex (100 µg/kg) in suppressing clinical score presentation. However, whilst high-dose Dex induced hyperglycaemia, neither the optimal co-therapy nor its constituents alone, altered blood glucose significantly (Figure 4A). Furthermore, we found that eCT (1.0 µg/kg) co-administration reduced high-dose Dex-induced hyperglycaemia by 48% (Figure 4A).

In line with the hyperglycemia data, eCT did not worsen the suppression of blood ACTH levels produced by Dex, actually trending towards attenuation, irrespective of whether it was used at a sub-therapeutic (15 µg/kg) or fully therapeutic (100 µg/kg) dose (Figure 4B).

**Figure 5:**
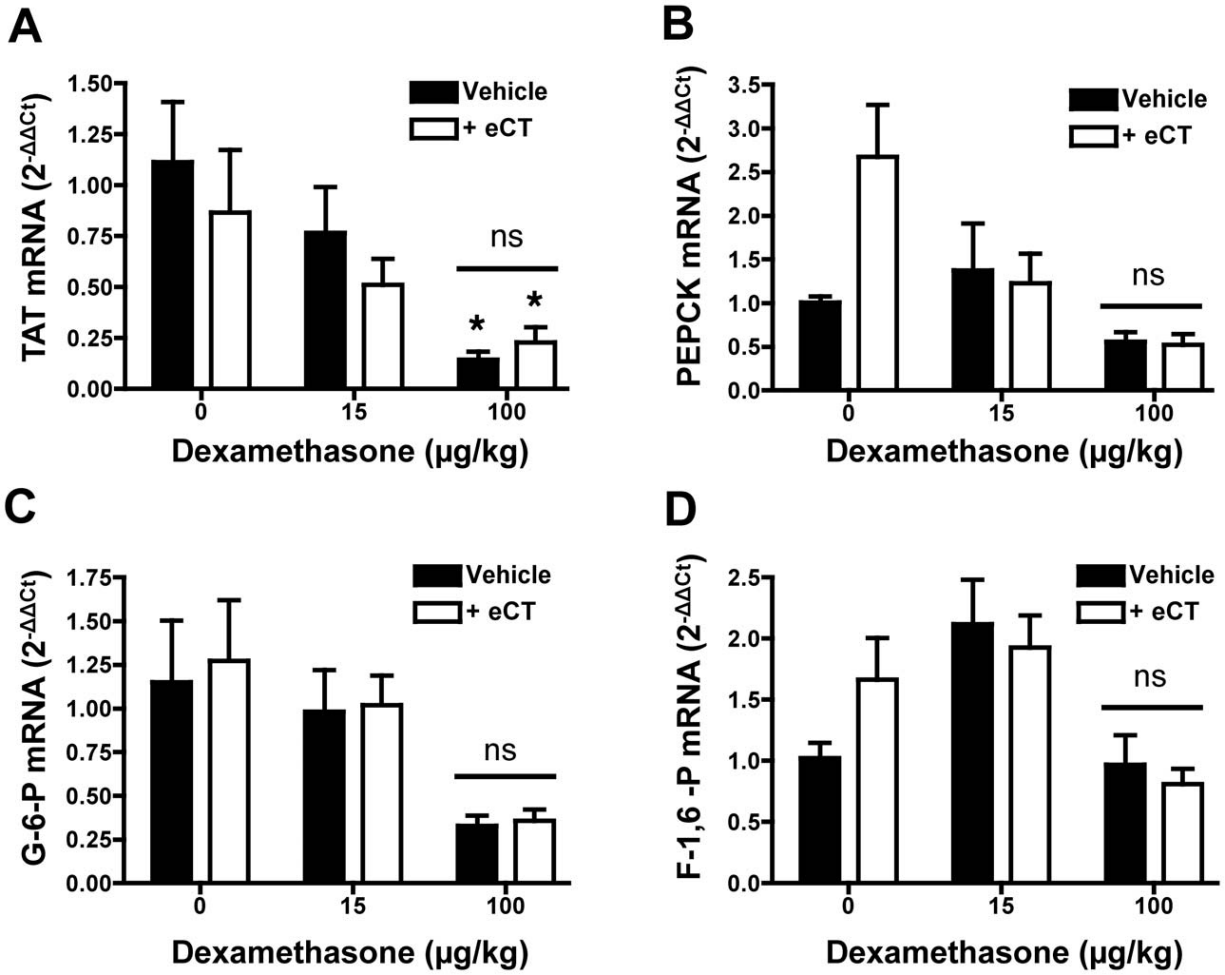
Elcatonin co-administration does not augment Dexamethasone-induced changes in gluconeogenesis-related liver enzyme mRNA. Quantitative real time-PCR was performed in liver tissues harvested from the hyperglycaemia experiment (see Legend to Figure 4 for protocol), in which fasted rats received single dosing of Dexamethasone (µg/kg) with or without elcatonin (eCT; 1.0 µg/kg). The expression of the following genes was studied: (A) tyrosine aminotransferase (Tat); (B) phosphoenolpyruvate carboxykinase (Pck2); (C) glucose-6-phosphatase (G6pc3); (D) fructose-1,6-bisphosphase (Fbp2). Data was expressed as 2^−ΔΔCt^ using Gapdh as endogenous control. Values report mean ± SEM of 4 distinct rat samples. Statistical analyses by one-way ANOVA (Kruskal-Wallis test with Dunn’s post-test); *p<0.05, ns p>0.05 as compared to vehicle-treated group.

Liver samples were collected from the animals used in the hyperglycaemia protocol to monitor modulation of genes coding for gluconeogenesis-related enzymes. There were several changes in mean values, however only the reduction of Tat mRNA by high-dose Dex was significant (Figure 5A). Importantly, eCT did not significantly alter mRNA levels, and co-administration had no significant impact upon Dex-induced changes (Figure 5A to D).

Liver samples from CIA experiments were also used for gluconeogenesis enzyme message quantification. Analysis was performed on ‘efficacy bands’ comprising treatment groups paired by their therapeutic outcome; i.e. the ‘moderate efficacy band’ comprises [Dex 30 µg/kg] and [Dex 7.5 µg/kg+eCT 1.0 µg/kg] regimens which collectively achieve ∼70% clinical score reduction, whilst the ‘high efficacy band’ comprises [Dex 100 µg/kg] and [Dex 15 µg/kg+eCT 1.0 µg/kg] which afford ∼100% efficacy. Three key observations arose from this analysis. Elcatonin alone significantly increases Tat mRNA in the CIA context (Figure S5A). In the high efficacy band, the co-therapy results in a significantly greater level of mRNA for Tat, Pck2 and G6pc3 (Figure S5A to C). Fbp2 exhibited a markedly different pattern to the other three targets. Dex, in the context of rat CIA, increases Fbp2 gene transcription in a dose-dependent manner. The co-therapy abolished elevations of Fbp2 mRNA in the moderate and high efficacy bands (Figure S5D).

## Discussion

With this study we report a series of in vivo and ex vivo analyses to reveal the biological properties of the eCT/Dex combination. Together with our previous report on salmon CT and prednisolone [20], these new data support the existence of a class-effect CT/GC synergism. Importantly, we pinpointed specific effectors of the synergism, with CXCL5 identified as a novel potential marker for this anti-inflammatory treatment in both tissue and plasma. Collectively, these preclinical analyses provide the rational for testing a GC and a CT preparation as a novel co-therapy for chronic human inflammatory pathologies.

A major aim of this study was to characterise an optimal CT/GC combination. Elcatonin alone was not therapeutic in rat CIA, confirming previous data with salmon CT [20] and making this model of arthritis ideal for stretching the co-therapy to its limit of efficacy. We found this limit to lie at the intersection between 15 µg/kg Dex and 1.0 µg/kg eCT, which afforded an abolition of clinical score presentation with an 85% GC dose reduction. Equally, a combination therapy of 7.5 µg/kg Dex and 1.0 µg/kg eCT, given daily from disease onset, produced an effect similar to that attained by a four-fold higher dose of Dex. It should be noted that a tight therapeutic window emerged with respect to the eCT dose and the synergistic outcome, with the dose of 1 µg/kg eCT giving consistent synergy throughout the study.

The integrity of the joint in the animals treated with the effective co-therapy, evident at the macroscopic level, was confirmed histologically, with a clear absence of pannus formation, very low degree of immune cell infiltrates into the synovial tissue and virtual absence of erosion into the cartilage/bone. The latter effect is reminiscent of the effects of salmon CT [20] and could be corroborated by the negative modulation afforded by eCT alone on circulating CTX-I. TRAP-5b is a marker of osteoclast number, rather than osteoclast activity [34], with both diagnostic and prognostic applications in osteoporosis and other diseases involving bone resorption [35]. It is worth noting that MMP-2 is secreted by cultured rheumatoid synovial fibroblasts [36], and is elevated in the synovial fluid and serum of RA patients [37], [38]. Synergistic modulation of these two players associated with articular disease provides mechanistic support to the therapeutic potential of the co-therapy here proposed.

Equally important observations could be evinced from the protein array. Whilst the dot blot array indicated a few false positives, as revealed by quantitative and specific ELISA determinations, the identification of CXCL5 may become of great importance. This CXC chemokine is secreted by RA synoviocytes and accounts for ∼40% of the neutrophil chemoattractant capacity of RA synovial fluid in vitro [39]. In addition, in co-culture assays, synovial fibroblasts release CXCL5 which is then exposed on endothelial cells to attract immune cells [40]. Several studies have linked GCs to CXCL5 attenuation [41]–[43] and, indeed, this chemokine was first identified as an LPS-induced GC-attenuated response gene product [44]. No literature to our knowledge directly relates CXCL5 to CT.

In view of the biological properties of CXCL5, its reduction in the joint extracts provides a novel mechanism of action for the co-therapy; however, of equally great importance is the evident synergism for reduction in circulating CXCL5 with the co-treatment Dex 7.5 µg/kg+eCT 1.0 µg/kg, giving values in arthritic rats similar to those of non-arthritic naïve animals. Altogether, these data prompt us to propose that inhibition of CXCL5 generation in the joint may be one of the mechanistic effectors of the synergism, and its modulation in the circulation could be exploited as a reliable biomarker for the clinical development of the co-therapy.

In the final part of the study we addressed the important issue of side effects: would the co-therapy result in a synergistic augmentation of canonical side effects characteristic of GC therapeutic use? The acute administration of eCT together with low-dose Dex had no effect on changes in glycaemia (in fasting rats) and circulating ACTH indicating that CT does not synergise with the glucocorticoid in relation to these outcomes; in contrast, at full dosage of Dex, eCT seems to attenuate, and certainly not to augment, these side effects.

Affirmatively, eCT co-administration did not alter acute Dex-induced modulation of liver enzymes, and, when comparing regimens by their anti-arthritic efficacy, eCT effectively abolished the Dex-induced elevation of liver F-1,6-BP mRNA in CIA. However, more intriguing were the results obtained for the expression of the other liver enzymes in CIA. When plotted against efficacy, Tat mRNA assessed in liver tissue from CIA rats treated with the co-therapy versus Dex alone revealed an intersection between apparent bell-shaped and inverse bell-shaped profiles (Figure S5A). Elcatonin significantly increased Tat message alone or in combination with 15 ug/kg Dex, but not with 7.5 ug/kg Dex. The Pck2 and G6pc3 data also partially support this indication (Figure S5B, C). This finding is, of course, not entirely surprising since CT alone can induce hepatic gluconeogenesis [45]–[47]. The important point to be made here is that neither agent alone is the magic bullet. Any ‘magic’ is to be found in a truly optimised combination. Our data supports the assertion that an appropriately optimised CT/GC synergism appears to be specific to the desirable therapeutic effects in the context of experimental arthritis.

GCs have presented us with one of medicine’s truly canonical perplexities. Their clinical utility renders these agents indispensable, yet the severity of adverse effects cannot be disregarded. An awareness of both their benefits and risks has been accumulating in the literature since the 1950’s. Since then, voluminous efforts to separate benefit from risk, *via* pharmacokinetic and dissociation strategies, have not afforded established clinical translation. Thus, there has long been, and remains, a pressing need for an adequate solution. An NO-donor GC analogue (‘nitro-steroid’) has been found to offer an enhanced anti-inflammatory and anti-arthritic effect in rodent models [48], while preventing GC-induced hypertension [49]; and a prednisolone/dipyridamole combination also provides greater anti-inflammatory effect in acute and chronic models, with reduced GC-induced HPA axis suppression and Tat induction [50] – a combination which is currently in phase II development for RA.

### Conclusions

Therapeutic GC doses bring the burden of unwanted side effects as a consequence of broad genomic effects, and countering efforts have prioritised the dissection of this reality, to rescue the strengths from the weaknesses. However, it is our assertion that the true strength of GCs lies within this apparent weakness. GCs may be particularly amenable to co-therapeutic synergy by virtue of their broad genomodulatory action – recasting them as a minefield of latent synergies. The combination of low- and even sub-therapeutic GC doses with suitable candidates can afford enhanced therapeutic effect. Optimally these co-therapies also bring a diminution of the classical GC adverse effects, either indirectly *via* a dose reduction of the GC, or *via* a directly effect of the co-therapy agent. Thus our recommendations are two-fold, that our evidence warrants clinical development of the CT/GC co-therapy and that our evidence be taken as a call for a generalised transition of thought regarding the optimal utility of GC agents. It is our belief, that by transitioning from a magic bullet monotherapy paradigm to an arguably more elegant harnessing of latent synergy potentials we can finally, not solve the GC ‘problem’, but transcend it.

## Supporting Information

Figure S1 **Collagen-induced arthritis in the female Lewis rat reaches 100% incidence.** Mean±SEM percentage incidence as assessed by positive clinical score presentation across twelve separate experiments (n = 76 to 82, except for day 21 where n = 16).
(TIF)

Figure S2 **Elcatonin synergises with Dex in the rat CIA model.** Time course of exemplary CIA experiment in rats treated with collagen on day 0. At the first signs of disease (Day 11), vehicle, Dexamethasone (7.5 µg/kg) or Dexamethasone plus the reported doses of Elcatonin were given daily i.p.; clinical score was monitored for a further week up to Day 18. Data are mean±SEM (n = 7 rats per group).
(TIF)

Figure S3 **Cytokine proteome profiler indicates potential markers in tissue extracts.** (**a**) Profile from pooled vehicle-treated group, n = 7. **(b)** Profile from pooled co-therapy (eCT 1.0 µg/kg+Dex 7.5 µg/kg) group, n = 7. Legend grid highlights analytes of greater expression.
(TIF)

Figure S4 **CCL5, CCL20 and CXCL7 are not markers of anti-arthritic treatment.** (**a**) CCL5, (**b**) CCL20 and **(c)** CXCL7 in paw tissue extracts from day 18 as determined by ELISA. Levels are expressed as Mean±SEM analyte mass per milligram of total protein. Co-Tx denotes the combination of Dex 7.5 µg/kg and eCT 1.0 µg/kg. (n = 6 to 14).
(TIF)

Figure S5 **The effect of eCT co-administration on Dex-induced liver enzyme message in the CIA model.** RNA extraction and RT-PCR was performed using liver tissue from the CIA protocol (see Legend to Figure 1), harvested on day 18. Data are mean±SEM 2eΔΔCt of 4 rats for (A) TAT, (B) PEPCK, (C) G-6-P and (D) F-1,6,BP plotted against the efficacy of each regimen (% clinical score reduction). Statistical analyses by Mann-Whitney test; *p<0.05 in single comparisons between regimens paired by efficacy band (anti-arthritic effect of: 0%, 65–75% and 90–100%).
(TIF)
