# Supplementary figures and images for: The Calcitonin and Glucocorticoids Combination: Mechanistic Insights into Their Class–Effect Synergy in Experimental Arthritis
Source: PLoS One. 2013 Feb 5;8(2):e54299. doi: 10.1371/journal.pone.0054299 (PMC3564948; doi:10.1371/journal.pone.0054299)

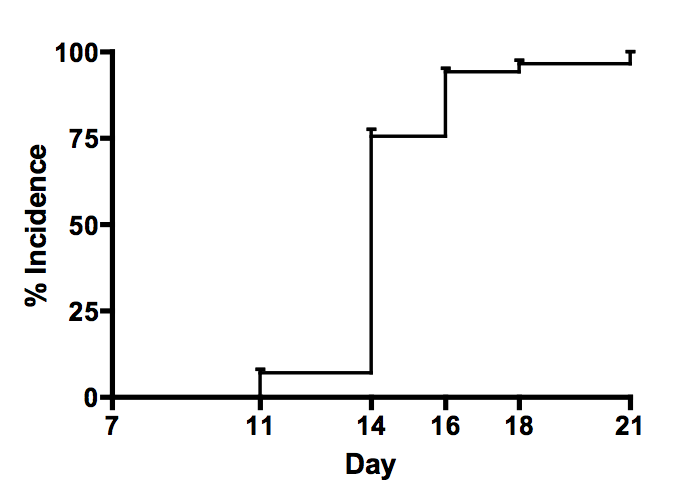

Supplement: Figure S1 — Collagen-induced arthritis in the female Lewis rat reaches 100% incidence. Mean±SEM percentage incidence as assessed by positive clinical score presentation across twelve separate experiments (n = 76 to 82, except for day 21 where n = 16). (TIF) [file pone.0054299.s001.tif]

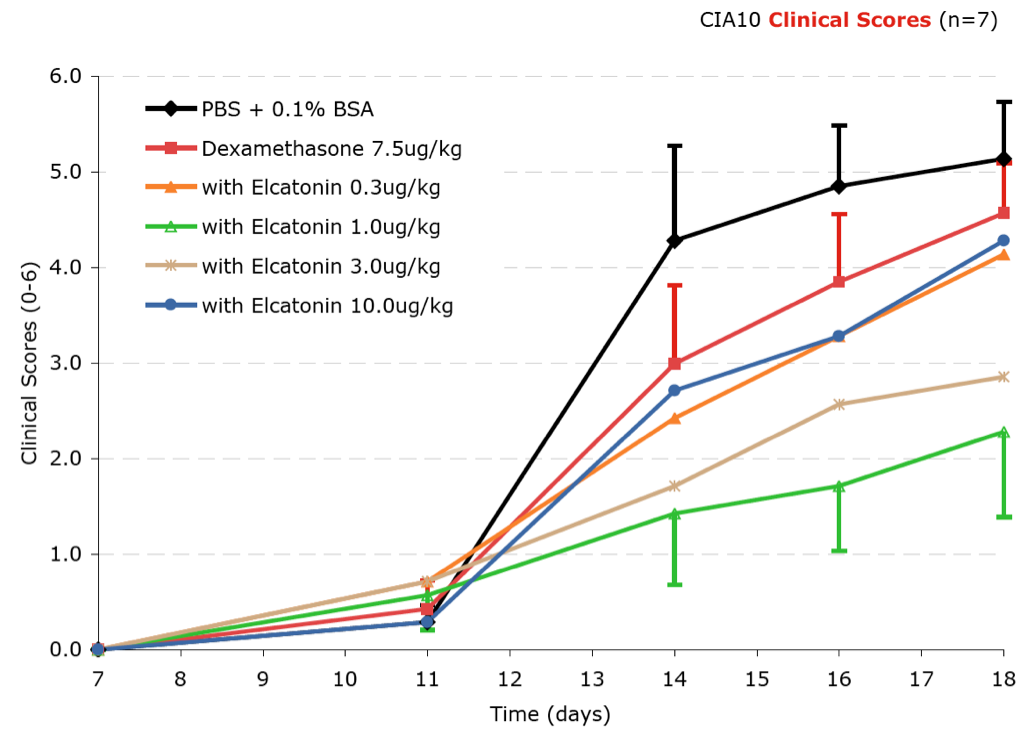

Supplement: Figure S2 — Elcatonin synergises with Dex in the rat CIA model. Time course of exemplary CIA experiment in rats treated with collagen on day 0. At the first signs of disease (Day 11), vehicle, Dexamethasone (7.5 µg/kg) or Dexamethasone plus the reported doses of Elcatonin were given daily i.p.; clinical score was monitored for a further week up to Day 18. Data are mean±SEM (n = 7 rats per group). (TIF) [file pone.0054299.s002.tif]

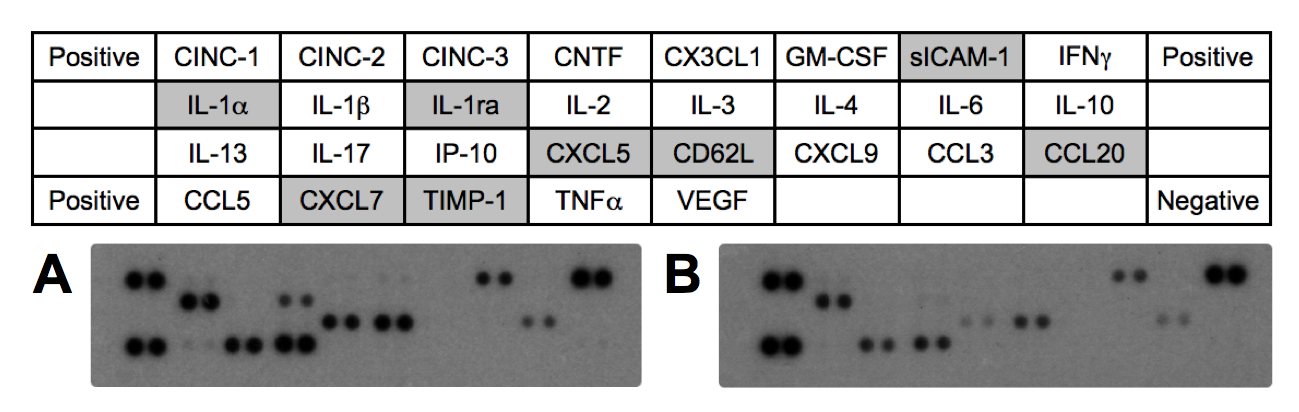

Supplement: Figure S3 — Cytokine proteome profiler indicates potential markers in tissue extracts. (a) Profile from pooled vehicle-treated group, n = 7. (b) Profile from pooled co-therapy (eCT 1.0 µg/kg+Dex 7.5 µg/kg) group, n = 7. Legend grid highlights analytes of greater expression. (TIF) [file pone.0054299.s003.tif]

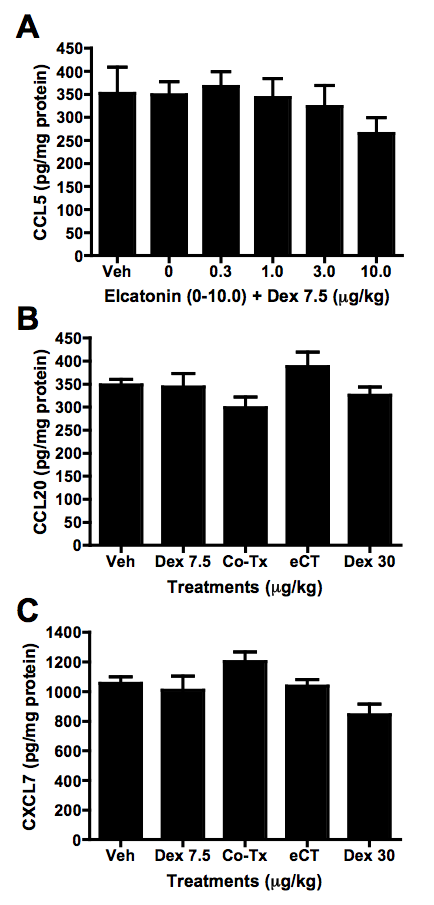

Supplement: Figure S4 — CCL5, CCL20 and CXCL7 are not markers of anti-arthritic treatment. (a) CCL5, (b) CCL20 and (c) CXCL7 in paw tissue extracts from day 18 as determined by ELISA. Levels are expressed as Mean±SEM analyte mass per milligram of total protein. Co-Tx denotes the combination of Dex 7.5 µg/kg and eCT 1.0 µg/kg. (n = 6 to 14). (TIF) [file pone.0054299.s004.tif]

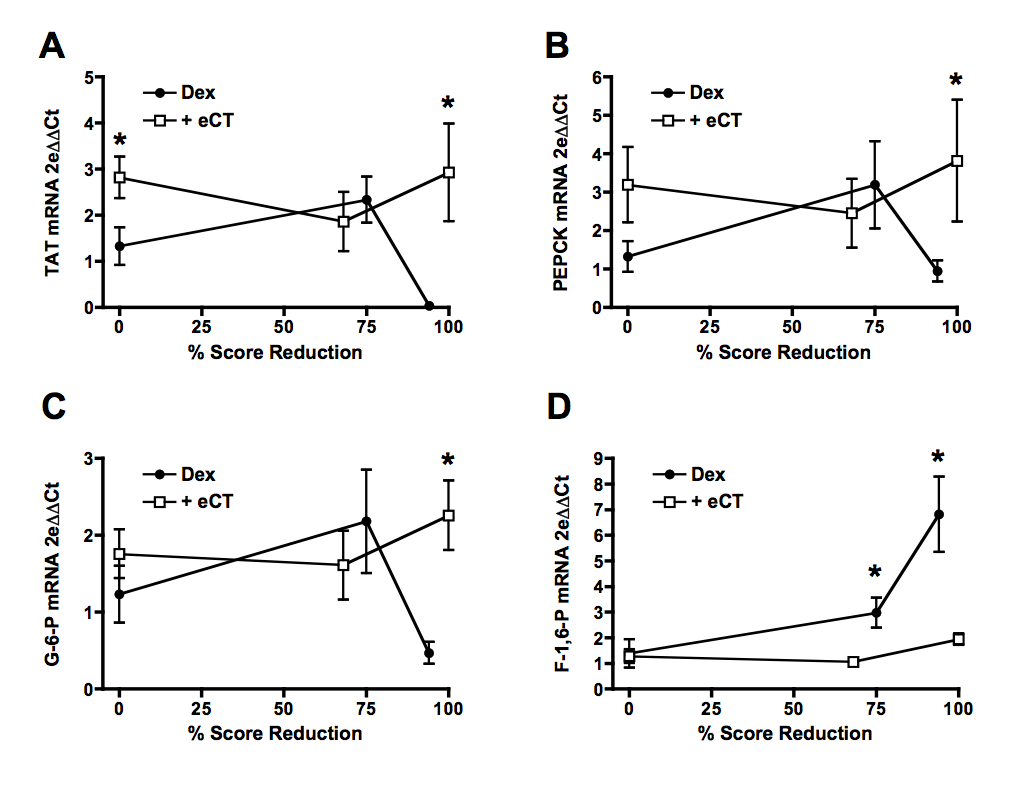

Supplement: Figure S5 — The effect of eCT co-administration on Dex-induced liver enzyme message in the CIA model. RNA extraction and RT-PCR was performed using liver tissue from the CIA protocol (see Legend to Figure 1), harvested on day 18. Data are mean±SEM 2eΔΔCt of 4 rats for (A) TAT, (B) PEPCK, (C) G-6-P and (D) F-1,6,BP plotted against the efficacy of each regimen (% clinical score reduction). Statistical analyses by Mann-Whitney test; *p<0.05 in single comparisons between regimens paired by efficacy band (anti-arthritic effect of: 0%, 65–75% and 90–100%). (TIF) [file pone.0054299.s005.tif]
